# *BRCA1* And *BRCA2* analysis of Argentinean breast/ovarian cancer patients selected for age and family history highlights a role for novel mutations of putative south-American origin

**DOI:** 10.1186/2193-1801-1-20

**Published:** 2012-09-25

**Authors:** Angela Rosaria Solano, Gitana Maria Aceto, Dreanina Delettieres, Serena Veschi, Maria Isabel Neuman, Eduardo Alonso, Sergio Chialina, Reinaldo Daniel Chacón, Mariani-Costantini Renato, Ernesto Jorge Podestá

**Affiliations:** 1 Laboratorio HRDC, INBIOMED-CONICET, Departamento de Bioquímica Humana, Facultad de Medicina, Universidad de Buenos Aires, Paraguay 2155 Piso 5, (C1121ABG) Buenos Aires, Argentina; 2 Department of Clinical and Experimental Sciences, “G. d’Annunzio” University, Via dei Vestini 1, 66100 Chieti, Italy; 3 Centro de Estudios Médicos e Investigaciones Clínicas, Galván 4102, (C1431FWO) Buenos Aires, Argentina; 4 Unit of General Pathology, Department of Medical, Oral and Biotechnological Sciences, “G. d’Annunzio” University, and Aging Research Center (CeSI), G. d’Annunzio Universith Foundation, Via Colle dell’Ara, 66100 Chieti, Italy; 5 Hospital Italiano, Virasoro 1249 (CB1921XAA), Rosario, Santa Fé, Argentina

**Keywords:** Argentina, early onset breast cancer, *BRCA1/BRCA2*, Germline mutations, Genetic variants, Familial breast cancer, Ashkenazi, Ethnicity

## Abstract

**Background:**

The spectrum of BRCA1/2 genetic variation in breast-ovarian cancer patients has been scarcely investigated outside Europe and North America, with few reports for South America, where Amerindian founder effects and recent multiracial immigration are predicted to result in high genetic diversity. We describe here the results of *BRCA1/BRCA2* germline analysis in an Argentinean series of breast/ovarian cancer patients selected for young age at diagnosis or breast/ovarian cancer family history.

**Methods:**

The study series (134 patients) included 37 cases diagnosed within 40 years of age and no family history (any ethnicity, fully-sequenced), and 97 cases with at least 2 affected relatives (any age), of which 57 were non-Ashkenazi (fully-sequenced) and 40 Ashkenazi (tested only for the founder mutations c.66_67delAG and c.5263insC in *BRCA1* and c.5946delT in *BRCA2*).

**Discussion:**

We found 24 deleterious mutations (BRCA1:16; BRCA2: 8) in 38/134 (28.3%) patients, of which 6/37 (16.2%) within the young age group, 15/57 (26.3%) within the non-Ahkenazi positive for family history; and 17/40 (42.5%) within the Ashkenazi. Seven pathogenetic mutations were novel, five in *BRCA1*: c.1502_1505delAATT, c.2626_2627delAA c.2686delA, c.2728 C > T, c.3758_3759delCT, two in *BRCA2*: c.7105insA, c.793 + 1delG. We also detected 72 variants of which 54 previously reported and 17 novel, 33 detected in an individual patient. Four missense variants of unknown clinical significance, identified in 5 patients, are predicted to affect protein function. While global and European variants contributed near 45% of the detected *BRCA1/2* variation, the significant fraction of new variants (25/96, 26%) suggests the presence of a South American genetic component.

This study, the first conducted in Argentinean patients, highlights a significant impact of novel BRCA1/2 mutations and genetic variants, which may be regarded as putatively South American, and confirms the important role of founder BRCA1 and BRCA2 mutations in Argentinean Ashkenazi Jews.

**Electronic supplementary material:**

The online version of this article (doi:10.1186/2193-1801-1-20) contains supplementary material, which is available to authorized users.

## Introduction

Hereditary breast cancer accounts for 5-10% of all BC cases [1] and is characterized by dominant inheritance, premenopausal diagnosis, more severe course, bilaterality and frequent association with ovarian cancer (OC) [2]. The identification of the two major hereditary breast/ovarian cancer genes, *BRCA1* (17q21, MIM* 113705) in 1994 [3] and *BRCA2* (13q14, MIM* 600185) in 1995 [4], led to a new era in the diagnosis of inherited high predisposition to breast and ovarian cancer [5, 6]. Breast-ovarian cancer (BOC)-causing mutations and other genetic variants are distributed along the entire coding and non-coding regions of *BRCA1* and *BRCA2*, and more than 3400 gene variants have been described in the *Breast Cancer Information Core* (BIC) [7]. New variants continue to be detected worldwide, mostly in *BRCA1.*

The prevalences of *BRCA1/BRCA2* mutations in BOC patients with early onset (EO) and/or BOC family history (FH) appear to be similar across race/ethnicity, but there is evidence of important racial and/or geographic differences in the spectrum of *BRCA1/2* genetic variation, including pathogenic mutations and variants of uncertain significance. These differences may reflect population history and genetic drifts, and could have a significant impact on genetic counselling, genetic testing, and follow-up care [8]. A typical example is provided by the case of Ashkenazi Jews, where three founder mutations: *BRCA1* c.66_67delAG *BRCA1* c.5263insC, and *BRCA2* c.5946delT account for most of familial breast-ovarian cancer [9]. Founder *BRCA1* and *BRCA2* mutations in Ashkenazi Jews in Israel: frequency and differential penetrance in ovarian cancer and in breast-ovarian cancer families [10].

*BRCA1/2* mutation status in subsets of BOC patients selected for age, BOC family history and ethnicity has been scarcely investigated outside Europe and North America [5, 11–15], with few reports for South America, where Native American founder effects and the complex multiracial demography of recent immigration are predicted to result in high genetic variation[16]. Indeed, recent studies point to a role of Native American ancestry in *BRCA1/2* disease patterns in Central and Northern America [17–22]. Epidemiological data indicate that in Argentina BC incidence [23] and mortality rates [24] are among the highest in the world. The historical records and epidemiological and molecular studies point to variable degrees of admixture among European, mainly Spanish and Italian, and Native American components in more than 50% of the Argentinean population [16, 25]. Regarding autosomal evidence of admixture, the relative European, Native American, and West African genetic contributions to the Argentinean gene pool were estimated to be 67.55%, 25.9%, and 6.5%, respectively [7].

Our study is the first report describing *BRCA1/BRCA2* gene variants in Argentinean BOC patients, and highlights a significant impact of novel mutations and genetic variants which may be regarded as putatively South American. On the other hand, we confirm the key role of founder *BRCA1* and *BRCA2* mutations in Argentinean Ashkenazi Jews.

## Methods

The study includes 134 BOC probands selected either for age at cancer diagnosis or for family history (FH), according to the criteria listed in Table 1. The patients selected for diagnosis within 40 years of age and no BOC FH (EO patients, any ethnicity) included 37 cases (21 with BC, 13 with OC, 3 with BOC; age range 12–40 years, mean age 31.0 ± 7.5 years). The FH patients (any age, 97 cases overall), selected based on the presence of at least two BOC-affected 1^st^ or 2^nd^ degree relatives, included 57 non-Ashkenazi patients (32 with BC, 18 with OC, 7 with BOC, age range 26–71 years, mean 44.6 ± 10.9 years), and 40 Ashkenazi patients (32 with BC, 6 with OC and 2 with BOC, age range: 32–64 years, mean age 47.1 ± 9.9 years) (Tables 1 and 2). The Ashkenazi subset was tested only for the panel of the three founder Ashkenazi mutations (c.66_67delAG (reported in BIC as 185delAG), and c.5263insC (in BIC as 5382insC) in *BRCA1* and c.5946delT (in BIC as 6174delT) in *BRCA2*); all the other cases were fully sequenced.

**Table 1:** **Inclusion criteria for the probands**

Group (n)	Criteria	Number of probands
EO (37)	Onset of cancer ≤40 years	37
Ashk-FH (40)	Onset of cancer ≤40 years with family history	12
	Onset of cancer >40 years with family history	28
FH (57)	Onset of cancer ≤40 years with family history	31
	Onset of cancer >40 years with family history	26
Total		134

**n**: total number of probands per group. **EO**: Early onset; **Ashk**: Ashkenazi; **FH**: Family history, defined as: at least 2 members of 1^st^ or 2^nd^ degree with breast and/or ovarian cancer.

**Table 2:** **Summary of the mutations detected**

Total patients = 134 (group)	Age at diagnosis (n)	Family history	***BRCA1***mutation (%)	***BRCA2***mutation (%)	% of mutated
37 (EO)	≤40 years (37)	No	4 (10.8)	2 (5.4)	16.2
40 (Ashk-FH)	≤40 years (12)	Yes	3 (25.0)	4 (33.3)	58.3
	>40 years (28)	Yes	6 (21.4)	4 (14.3)	35.7
57 (FH)	≤40 years (31)	Yes	3 (9.7)	3 (9.7)	19.4
	>40 years (26)	Yes	7 (26.9)	2 (7.7)	35.8

**EO**: Early onset; **Ashk**: Ashkenazi; **FH**: Family History.

Total coding *BRCA1-2* sequencing was performed for the patients in all groups except.

the Ashkenazi patients which were tested for the panel of three mutations.

**(n):** number of probands analyzed.

Blood samples were sent from the participating centers to the Laboratory HRDC of the Department of Biochemistry, University of Buenos Aires, and were also recruited at the Centro de Estudios Medicos e Investigaciones Clinicas (CEMIC). Study eligibility required signing an informed consent as a result of the routine procedures for genetic analysis. This study was approved by the Ethics Committee of the *Sociedad Argentina de Investigación Clínica.*

Genomic DNA was isolated using the QIAamp DNA blood purification kit (Qiagen, http://www.qiagen.com). The coding sequences and exon-intron boundaries of the *BRCA1-2* genes were analyzed by amplification using polymerase chain reaction (PCR) with alternative primers to avoid false results due to polymorphisms [26, 27], followed by direct sequencing of at least 55 amplicons, to ensure overlapping of the segments. Sequencing was performed using either an Applied Biosystems 3730xl DNA Analyzer or an Applied Biosystems ABI PRISM ® 310 Genetic Analyzer. Homozygosis (HO) was confirmed by alternative sequencing in exonic and/or intronic regions. The three Ashkenazi mutations were tested as described [28]. Variants nomenclature follows the guidelines of the Human Genome Variation Society (HGVS). Tables provide lists including also the nomenclature of the Cancer Information Core Internet Website (BIC), April 2012.

Effects of the missense mutations that resulted not reported or recorded as clinically unknown (CU) in the BIC were predicted by virtual analyses of functional compatibility for aminoacid changes using two programs: Align-GVGD (http://agvgd.iarc.fr/) [29] and SIFT (http://sift.bii.a-star.edu.sg/) [30].

## Results and discussion

We describe for the first time in Argentina the results of *BRCA1/BRCA2* germline analysis in 134 BOC probands selected either for diagnosis within 40 years of age (37 cases) or for FH (97 cases) (Tables 1 and 2). The latter included 40 Ashkenazi patients, tested only for the three founder Ashkenazi mutations [28]. All the other cases were fully sequenced.

Overall 96 mutations and sequence variants, of which 53 in *BRCA1* and 43 in *BRCA2,* were identified in 94/134 patients analyzed. Mutation types, effects, carrier frequencies, worldwide occurrences and relevant references are listed in online Additional file 1: Tables S1 and Additional file 2: Table S2. The sequence variants were classified as pathogenic based on literature data and/or when predicted to truncate/inactivate the protein product.

Among the 53 sequence variants identified in *BRCA1* 15 are novel and 17 clinically unknown, 14 introduce a stop codon; 22 are missense substitutions (Additional file 1: Table S1). With regard to the 43 *BRCA2* mutations, 9 are novel, 17 clinically unknown, 6 introduce a stop codon; 15 are missense substitutions and one is predicted to result in an aberrant splice (Additional file 2: Table S2). The truncating mutations and the novel non-truncating variants predicted to affect the *BRCA1* and *BRCA2* gene products are described in Table 3. Synonyms, intronic and polymorphic *BRCA1* and *BRCA2* variants ranged from 4 to 33 per individual patients and were detected in all the 94 fully-sequenced cases (Additional file 1: Table S1 and Additional file 2: Table S2). Notably, 34 variants are listed in BIC as of clinically unknown importance, and of these 14 were identified in unique patients (Additional file 1: Table S1 and Additional file 2: Table S2).

**Table 3:** ***BRCA1/BRCA2*truncating mutations, novel and non-truncating variants affect the gene products**

***Exon***	***Codon***	***(HGVS) Protein level***	***(HGVS) DNA level***	***BIC DNA level***	***BIC Status***	***Carrier CODE***	***Index case Status (age)***	***Family history***	***Inclusion Criteria***	***Worldwide Occurrence***
***BRCA1***										
2	E23VfsX16	Stop cod39	c.66_67delAG	185delAG	D	AB54	Br(37)	Br	Ashk-EO-FH	Ashkenazi
						AB60	Br(40)	Br	Ashk-EO-	
						AB77	Ov (44)	Br, Ov	FH Ashk-	
						AB68	Ov (44)	Br, Ov	FH Ashk-	
						AB76	Br (49)	Br	FH Ashk-	
						AB81	Br (52)	Br	FH Ashk-	
						AB87	Ov (60)	Br, Ov	FH Ashk-FH	
2	E23KfsX18	Stop cod40	c.67insA	186insA	D	AB82	Br (34)	Br	EO-FH	NE/ME
5	C61G	p. Cys61Gly	c.181 T > G	300 T > C	D	AB75	Br (49)	Br	FH	E
5	R71G	p. Arg71Gly	c.211A > G	330A > G	D	AB64	Br (43)	Br	FH	E
7	E143X	p. Glu143Stop	c.427 G > T	546 G > T	D	AB46	Br (33)	Br, Ov, Pa, Pr	EO-FH	E
11	S267KfsX19	Stop cod285	c.797_798delTT	916delTT	D	AB36	Br-Ov (46)	Br	FH	C, L-A E, N-A
**11**	**K501Kfs30**	**Stop cod530**	**c.1502_1505delAATT**	**1621delAATT**	**NR**	**AB20**	**Br (32)**	**No**	**EO**	**Argentina**
11	R504VfsX28	Stop cod531	c.1510delC	1629delC	D	AB40	Br (30)	Br	EO-FH	E
**11**	**E836GfsX2**	**Stop cod837**	**c.507_2508delAA**	**2626delAA**	**NR**	**AB67**	**Br (50)**	**Br**	FH	**Argentina**
**11**	**S896Vfs104**	**Stop cod999**	**c.2686delA**	**2805delA**	**NR**	**AB85**	**Br (55)**	**Br**	FH	**Argentina**
**11**	**Q910X**	**p. Gln910Stop**	**c.2728 C > T**	**2847C > T**	**NR**	**AB84**	**Ov (55)**	**Br, Ov, Co**	FH	**Argentina**
11	R1203X	p. Arg1203Stop	c.3607C > T	3726C > T	D	AB8	Ov (25)	No	EO	C, L-A
11	E1210RfsX8	Stop cod1218	c.3627insA	3746insA	D	AB21	Br-Ov (33)	No	EO	C, L-A, As
**11**	**S1253X**	**p. Ser1253Stop**	**c.3758_3759delCT**	**3877delCT**	**NR**	**AB17**	**Br (31)**	**No**	**EO**	**Argentina**
17	T1677IfsX2	Stop cod1678	c.5030_5033delCTAA	5149delCTAA	D	AB79	Br (51)*	Br, Ov, Pa, Pr	FH	E
20	S1755PfsX75	Stop cod1829	c.5263insC	5382insC	D	AB55	Br (49)	Br	Ashk-FH Ashk-EO-FH	E, Ashkenazi
						AB97	Br (38)	Br		
***BRCA2***										
**9**	**-**	**Splice defect**	**c.793 + 1delG**	**IVS9 + 1delG**	**NR**	**AB99**	**Br (31)**	**Br**	**EO-FH**	**Argentina**
11	N955KfsX5	Stop cod959	c.2808_2811delACAA	3036delACAA	D	AB78	Br (50)	Br	FH	E, L-A
11	S1982RfsX22	Stop cod2003	c.5946delT	6174delT	D	AB43	Br (32)	Br-male	Ashk-EO-FH	Ashkenazi
						AB47	Br (33)	Br-male	Ashk-EO-	
						AB69	Br/Ov (45)	Br-male	FH Ashk-FH	
						AB57	Br (39)	Br, Pa,	Ashk-EO-	
						AB71	Br (46)	Ov Pr,	FH Ashk-	
						AB74	Br (48)	Br	FH Ashk-	
						AB95	Br (36)	Br	FH Ashk-	
						AB96	Br (60)	Br	EO-FH Ashk-FH	
11	K1213X	p. Lys1213Stop	c.6037A > T	6265A > T	D	AB34	Br (40)	No	EO	E
11	S1882X	p. Ser1882Stop	c.5644C > G	5872C > G	D	AB117	Br (50)	Br, Pr	FH	E
11	Y1894X	Stop cod1894	c.5909insA	6137insA	D	AB92	Br (31)	Br	EO-FH	E
**14**	**E2369EfsX23**	**Stop cod2391**	**c.7105insA**	**7333insA**	**NR**	**AB98**	**Br (35)**	**Br**	**EO-FH**	**Argentina**
18	D2723H	p. Asp2723His	c.8169 G > C	8397 G > C	CU	AB31	Ov (38)	No	EO	E

**D,** deleterious; **CU**, clinically unknown importance; **NR,** Not Reported in *Breast Information Core database(BIC)*http://research.nhgri.nih.gov/bic/

**Global,** as defined in BIC or when reported in at least 3 continents ethnic groups in HapMap; **E**, European; **As**, Asian; **A-A** African-American; **L-A**, Latin American; **N-A**, Native-American; **A-C** America-Caucasian; **NE/ME**, Near Eastern/Middle Eastern;

The DNA sequence numbering of BRCA1and BRCA2 sequence variants is based on recomendations of the Human Genome Variation Society (HGVS, translation initiation codon ATG = 1) *BRCA1:genomic sequence:L78833; RNA sequence: U14680; BRCA2 genomic sequence: NW_001838072; RNA sequence: NM_001838072*

In bold, novel mutations not previously reported.

Overall, a total of 24 bona fide pathogenetic mutations, 16 in *BRCA1* and 8 in *BRCA2*, were detected in 38/134 cases (28.4%), including: a) 6/37 (16.2%) fully-sequenced patients in the group within 40 years of age; b) 15/57 (26.3%) fully-sequenced non-Ashkenazi FH patients; c) 17/40 (42.5%) Ashkenazi FH patients, analyzed for the three Ashkenazi mutations only (Table 2). The pathogenetic mutations were more frequent in *BRCA1* (23/38, 60.5%) than in *BRCA2* (15/38, 39.5%), which is in agreement with literature data [31].

The Ashkenazi-FH patients with age ≤40 years showed the highest frequency of pathogenetic *BRCA1* and *BRCA2* mutations, i.e., 58.3% (for *BRCA1* 16.7% in c.66_67delAG and 8.3% in c.5263insC and 33.3% for *BRCA2* c.5946delT), in agreement with literature frequencies [28, 32]. The detection rate of bona fide pathogenetic mutations in FH-negative probands selected for age within 40 years at diagnosis was 6/37 (16.2%). This falls within the 15-31% range reported in the literature for EO BOC with FH [33–36], but is in contrast with the lack of mutations reported in EO Chilean patients without FH [37]. The published data on the South American population [17, 37, 38] show lower rates of mutation detection, while in agreement with results from a study in the USA [31] and a large study in high risk Hispanic family from USA [39] and also with an study of Hispanic BOC from Colombia [40]. Differences in mutation detection rates might reflect divergences in the criteria of proband selection and in the methods of analysis. In fact, the other South American [37, 38] reports were based on indirect mutation detection methods and not on full sequencing; in contrast, we used direct sequencing of all the amplicons along the *BRCA1* and *BRCA2* coding sequences and exon-intron boundaries.

It may be of interest to compare the deleterious mutation rates of the young patients with no FH (16.2%) and of the FH cases of similar age (within 40 years) in the non-Ashkenazi and Ashkenazi groups (Table 3). Notably, a pathogenetic mutation was found in 6/31 (19.4%) non-Ashkenazi FH patients within 40 years of age (mean age 35.6 ± 4.8 years) and in 7/12 (58.3%) FH Ashkenazi cases within the same age cutoff (mean 35.6 ± 2.8 years). The recurrent Ashkenazi mutations were never detected in non-Ashkenazi probands. *BRCA1* c.66_67delAG, *BRCA1* c.5263insC and *BRCA2* c.5946delT were found in 7 (17.5%), 2 (5%) and 8 (20%) Ashkenazi probands, respectively. Interestingly, *BRCA2* c.5946delT was also found in a non-Ashkenazi FH patient who could recall a great grand mother of Ashkenazi origin. Conversely, only a non-Ashkenazi pathogenetic mutation (Asp2723His in *BRCA2*) was detected in one of 4 patients of Ashkenazi origin included in the subset selected for early diagnosis and no FH. This supports the full sequencing of EO Ashkenazi patients with no BOC FH.

With regard to disease association, the pathogenetic mutations in *BRCA1* occurred in 16/88 BC cases (18.2%), 5/24 OC cases (20.8%) and 2/22 BOC cases (9.1%), those in *BRCA2* in 13/88 BC cases (14.8%), 1/24 OC cases (4.2%) and 1/22 BOC cases (4.5%). As expected, OC was more frequent in *BRCA1* carriers (21.7% vs 6.7%), and BC in *BRCA2* carriers (86.6% vs 65.2%) [30].

Seven pathogenic mutations (18.4% of all the mutations detected) were putatively novel: 5 in *BRCA1* (21.7% for this gene), all with frameshifts generating stop codons in exon 11, and 2 *in BRCA2 (13*.3% for this gene), one with a frameshift at nt 2369, exon 14 (c7333 insA), the other (c.793 + 1delG) affecting the donor splicing site nucleotide at IVS + 1 delG in intron 9 (Table 3).

The frequency of the common non-pathogenic variants and synonyms was in agreement with that reported in the BIC. The mutations reported in BIC as CU that we detected in multiple patients as homozygous (in parenthesis number of cases) and/or in association with deleterious mutations, such as p. Gln356Arg, IVS7 + 36 C > T, IVS7 + 41 C > T, IVS14-63 C > G, and IVS18 + 66 G > A in *BRCA1* and p. Val2171Val (9), p. Ala2466Val (4), IVS8 + 56 C > T, IVS9 + 65delT, IVS10 + 12delT, and IVS11 + 80delTTAA (1) in *BRCA2* most probably represent non-pathogenic variants. Furthermore, based on prediction programs, homozygous status, detection in multiple unrelated patients and/or association with pathogenic mutations, 10 variants found in the present study and not reported in the BIC can be considered non pathogenic. These include p. Val122Asp, p. Gln139Lys, IVS7 + 38 T > C, IVS7 + 49 del 15 bp, in *BRCA1* and c*110 A > C at 3'UTR in *BRCA2* (two other novel *BRCA2* variants, i.e., IVS4 + 246 G > C and IVS4 + 364delT, located far from the end of the exon 4 are reported here only as heterozygosity markers).

Five of the 28 missense variants (Table 4) (i.e., p. Arg7Cys, p. Cys61Gly, p. Arg71Gly, p. Tyr179Cys, and p. Met1652Thr in *BRCA1*, p. Asp2723His in *BRCA2*) were predicted to have an impact on protein structure upon evaluation by SIFT and GVGD (Table 4). *BRCA1* p. Arg7Cys, differently from the other non-conservative variants, has a rather low prediction score and was found in two cases. The high prediction values for *BRCA1* p. Cys61Gly and *BRCA1* p. Arg71gly agree with their previously reported pathogenicity [41, 42] (Table 4). Few reported data are available for *BRCA2* p. Asp2723His [43]. *BRCA1* p. Met1652Thr, located in the BRCT tandem repeat region is predicted to result in a large volume change in rigid neighbourhood [44] but structural and functional assays show normal peptide binding specificity and transcriptional activity [45]. Tyr179Cys is also located in a highly conserved region and is listed as clinically importance unknown (CU) in BIC. Notably BRCA1 Tyr179Cys co-occurred with two other missense mutations, i.e., Phe486Leu and Asn550His, in an FH patient affected with pagetoid BC (AB80). These 3 mutations, already reported to occur together, may constitute a rare haplotype [46] [brca.iarc.fr/LOVD].

**Table 4:** ***BRCA1/2*missense variants identified in 94 (non Askenazi) Argentinean breast/ovarian cancer cases**

***HGVS :Protein: DNA***		***BIC: Status***	***N° Carrier (%)***	***Co-occurrence with deleterious***	***PredictionSIFT GVGD grade***		***refSNP***
***BRCA1***							
**p. Arg7Cys**	**c.19C > T**	**CU**	**2(1.1)**	-	**NT**	**C15**	rs144792613
**p. Cys61Gly**	**c.181 T > G**	**D**	**1 (1.1)**	-	**NT**	**C65**	-
**p. Arg71Gly**	**c.211A > G**	**D**	**1 (1.1)**	-	**NT**	**C65**	-
p. Val122Asp	c.365 T > A	NR	5 (5.3)	*BRCA2*	T	C0	-
p. Gln139Lys	c.415C > A	NR	6 (6.3)	-	T	C0	-
**p. Tyr179Cys**	**c.536A > G**	**CU**	**1 (1.1)**	*BRCA1* [30] (AB80)	**NT**	**C35**	rs56187033
p. Gln356Arg	c.1067A > G	CU	10 (10.6)	*BRCA1/BRCA2#*	T	C0	rs1799950
p. Phe486Leu	c.1456 T > C	CU	1 (1.1)	*BRCA1* [30] (AB80)	T	C0	rs55906931
p. Val525Ile	c.1573 G > A	CU	1 (1.1)	-	T	C0	rs80357273
p. Asn550His	c.1648A > C	CU	1 (1.1)	*BRCA1* [30] (AB80)	NT	C0	rs56012641
p. Asp693Asn	c.2077 G > A	CN	8 (8.5)	*BRCA1*	T	C0	rs4986850
p. Pro871Leu	c.2612C > T	CN	29 (30.9)	*BRCA1/BRCA2#*	T	C0	rs799917
p. Lys898Glu	c.2692A > G	CU	1 (1.1)	*BRCA2*	T	C0	rs80357420
p. Met1008Ile	c.3024 G > A	CU	1 (1.1)	*BRCA1* [30]	T	C0	rs1800704
p. Glu1038Gly	c.3113 G > A	CN	33 (35.1)	*BRCA1/BRCA2#*	T	C0	rs16941
p. Ser1040Asn	c.3119 G > A	CU	1 (1.1)	*BRCA1* [31]	T	C0	rs4986852
p. Asp1131Glu	c.3393C > G	NR	1 (1.1)	*BRCA2*	T	C0	-
p. Lys1183Arg	c.3548A > G	CN	34 (36.2)	*BRCA1/BRCA2#*	T	C0	rs16942
p. Ile1275Val	c.3823A > G	CU	8 (8.5)	-	T	C0	rs80357280
p. Glu1586Gly	c.4757A > G	NR	1 (1.1)	-	NT	C0	-
p. Ser1613Gly	c.4837A > G	CN	33 (35.1)	*BRCA1/BRCA#*	T	C0	rs1799966
**p. Met1652Thr**	**c.4955 T > C**	**CU**	**1 (1.1)**	-	**NT**	**C25**	rs80356968
***BRCA2***							
p. Tyr42Cys	c.125A > G	CU(BIC)	1 (1.1)	-	T	C0	rs4987046
p. Asn289His	c.865A > C	CN	5 (5.3)	*BRCA2*	NT	C0	rs766173
p. His372Asn	c.1114C > A	CN	24 (4.2)	*BRCA1/BRCA2#*	T	C0	rs144848
p. Arg858Ile	c.2578 G > T	NR	1 (1.1)	*BRCA2*	T	C0	-
p. Asn991Asp	c.2971A > G	CN	4 (4.2)	*BRCA2*	T	C0	rs1799944
p.Q1063K	c.3187C > A	NR	1 (1.1)	-	T	C0	-
p. Asp1420Tyr	c.4258 G > A	CN	1 (1.1)	*BRCA2* [32]	T	C0	rs28897727
p. Met1915Thr	c.5744 T > C	CU	1 (1.1)	-	T	C0	rs4987117
p. Ser2098Phe	c.6749C > T	CU	1 (1.1)	-	T	C0	rs80358867
p. Arg2108His	c.6323 G > A	CU	1.(1.1)	-	T	C0	rs35029074
p. Ala2466Val	c.7397C > T	CU	37 (39.4)	*BRCA1*/*BRCA2*#	NT	C0	rs169547
p. Asn2486Lys	c.7919 T > G	NR	1(1.1)	-	T	C0	-
p. Ile2490Thr	c.7469 T > C	CU	6 (6.3)	-	NT	C0	rs11571707
**p. Asp2723His**	**c.8167 G > C**	**CU**	**1 (1.1)**	-	**NT**	**C65**	rs41293511
p. Ile3412Val	c.10690A > G	CU	3 (3.2)	-	T	C0	rs1801426

**NR**, Not Reported **CU**, Clinically Unknown**; CN,** clinically not important, in *Breast Information Core database* (BIC), http://research.nhgri.nih.gov/bic/;

In bold missense predict deleterious; **NT,** Not Tolerated; **T**, Tolerated; Align-**GVGD grade** between C0 and C65; **Co-occurrence: #** two or more patients.

**Figure 1:**
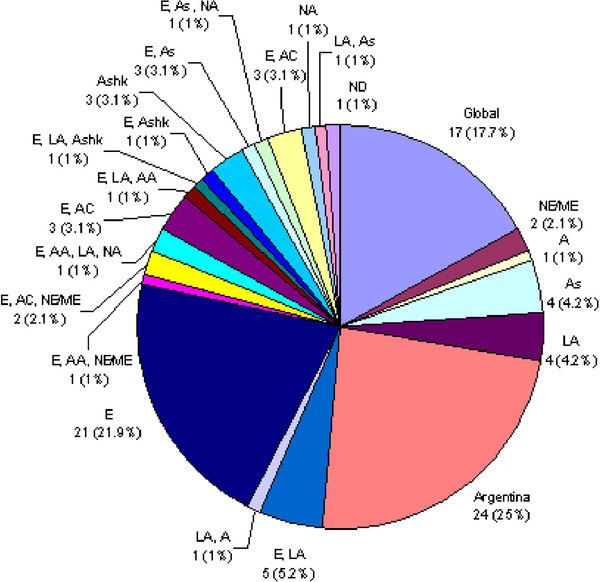
**Worldwide occurrence of 96 BRCA1/2 variants detected in 94 non-Ashkenazi Argentinean BOC patients.Global**, as defined in BIC or when reported in at least 3 continents in HapMap or in references; **A**, African; **AA**, African American; **AC**, American Caucasian; **As**, Asian; **Ashk**, Ashkenazi; **E**, European; **LA**, Latin American; **NA**, Native American; **NE/ME**, Near Eastern/Middle Eastern; **ND**, not determined.

In agreement with the complex population history of Argentina, the *BRCA1/2* mutations detected in this BOC series were associated with diverse geographic/ethnic backgrounds (Figure 1 and online Additional file 1: Table S1 and Additional file 2: Table S2). Of the 96 detected variants, 25 (26%) are not reported in the BIC, in HapMap [47] and in the current literature, and are thus putatively unique for Argentina, 17 (17.7%) were reported worldwide (at least 3 continents), 4 (4.2%) were reported only in Latin-America. The remaining variants comprise mutations previously detected in Europe, Asia and North America. (Additional file 1: Table S1 and Additional file 2: Table S2). The putative Latin American variants include p. Ile2490Thr in *BRCA2,* a modestly penetrant variant that might contribute to sporadic breast cancer risk, listed as CU in the BIC, originally described in a Caribbean patient [48] and reported almost 100 times in the BIC, frequently associated to Latin American probands. In this line, several novel variants were previously observed in Argentina in genes related to other hereditary syndromes and might be regarded as putatively regional or influenced by founding events, [49–51]. In the present case series this mutation recurred in six cases (Table 4, Figure 1)

Two other mutations related to South American ethnicity deserve mention: 1) *BRCA1* IVS7 + 37 del14bp(TTTTCTTTTTTTTT), not listed in BIC but found in 10/42 families from Uruguay (including one with a pathogenetic mutation in *BRCA2*) [38], and in a patient from Chile [37]; and 2) *BRCA2* IVS16-14 T > C, reported in a patient from Uruguay [38] and detected in 31 of our patients (including 4 with identified pathogenic mutations).

## Conclusions

The present study is the first reporting the spectrum of *BRCA1* and *BRCA2* mutations in an Argentinean BOC series, based on the analysis of the coding sequences and exon-intron boundaries of both genes. Given that the rates of BC incidence and mortality in Argentina are among the highest in the world [23, 24], a better understanding of the impact of *BRCA1/BRCA2*-related disease in Argentinean BOC patients is important for the implementation of prevention and/or early detection strategies. In our case series selected for early diagnosis with no FH or for FH independently of age at diagnosis, the overall detection rate of bona fide pathogenetic mutations was quite high (38/134, 28.3%). This could rise to 35.8% (48/134) including the missense mutations suspected to confer increasing risk of breast cancer.

Although global and European sequence variants contribute to near 45% of the detected *BRCA1/2* variation, the significant fraction of new variants putatively unique for Argentina detected in the present study might suggest the presence of a Native American genetic component, not yet genetically characterized, that it in recent centuries has come to admixture with alleles mostly of European origin.

## Electronic supplementary material

Additional file 1: **Table S1:***BRCA1* sequence variants identified in Argentinean breast/ovarian cancer cases [38, 52–57]. (DOC 25 KB)

Additional file 2: **Table S2:***BRCA2* sequence variants identified in Argentinean breast/ovarian cancer cases [58, 59]. (DOC 318 KB)

## Abbreviations

BOC: Breast/ovarian cancer
FH: Family history
BC: Breast cancer
OC: Ovarian cancer
BIC: Breast Cancer Information Core
EO: Early onset
PCR: Polymerase chain reaction
HO: Homozygosis
A: African
AA: American African
AC: American Caucasian
As: Asian
Ashk: Ashkenazi
E: European
LA: Latin American
NA: Native American
NE/ME: Near Eastern/Middle Eastern
ND: Not Determined
D: Deleterious
CU: Clinically Unknown
CN: Clinically No important
NR: Not reported
NT: Not tolerated
T: Tolerated
HGVS: Human Genome Variation Society.
