# Supplementary material for: BRCA1 And BRCA2 analysis of Argentinean breast/ovarian cancer patients selected for age and family history highlights a role for novel mutations of putative south-American origin
Source: Springerplus. 2012 Sep 25;1:20. doi: 10.1186/2193-1801-1-20 (PMC3725882; doi:10.1186/2193-1801-1-20)
Supplement: Supplementary file 2 — Additional file 2: Table S2:BRCA2 sequence variants identified in Argentinean breast/ovarian cancer cases [58, 59]. (DOC 318 KB) [file 40064_2012_24_MOESM2_ESM.doc]

**Table S1**: *BRCA1* sequence variants identified in Argentinean breast/ovarian cancer cases

| ***Location***  ***Exon*** | ***Codon*** | ***HGVS:***  ***Protein level*** | ***HGVS:***  ***DNA level*** | ***BIC:***  ***DNA level*** | ***BIC:***  ***Status*** | ***N° Carrier*** | ***Occurrence***  ***(Co-occurrence***  ***deleterious Mut or in ref)*** | ***SNP id*** | ***Worldwide***  ***Occurrence*** |
| --- | --- | --- | --- | --- | --- | --- | --- | --- | --- |
| ***Truncating*** | | | | | | | |  |  |
| 2 | E23VfsX16 | Stop cod 39 | c.66_67delAG | 185delAG | D | 7 | *BRCA1* (AB54, AB60, AB77, AB68, AB76, AB81, AB87) |  | Ashkenazi |
| 2 | E23KfsX18 | Stop cod 40 | c.67insA | 185insA | D | 1 | *BRCA1* (AB82) |  | NE/ME |
| 7 | E143X | p.Glu143Stop | c.427G>T | 546G>T | D | 1 | *BRCA1* (AB46) |  | E |
| 11 | S267KfsX19 | Stop cod 285 | c.797_798delTT | 916delTT | D | 1 | *BRCA1* (AB36) |  | L-A |
| **11** | **K501Kfs30** | **Stop cod 530** | **c.1502_1505delAATT** | **1621delAATT** | **NR** | **1** | *BRCA1* **(AB20)** |  | **Argentina** |
| 11 | R504VfsX28 | Stop cod 531 | c.1510delC | 1629delC | D | 1 | *BRCA1* (AB40) |  | E |
| **11** | **E836GfsX2** | **Stop cod 837** | **c.2507_2508delAA** | **2626delAA** | **NR** | **1** | *BRCA1* **(AB67)** |  | **Argentina** |
| **11** | **S896Vfs104** | **Stop cod 999** | **c.2686delA** | **2805delA** | **NR** | **1** | *BRCA1* **(AB85)** |  | **Argentina** |
| **11** | **Q910X** | **p.Gln910Stop** | **c.2728C>T** | **2847C>T** | **NR** | **1** | *BRCA1* **(AB84)** |  | **Argentina** |
| 11 | R1203X | p.Arg1203Stop | c.3607C>T | 3726C>T | D | 1 | *BRCA1* (AB8) |  | E, L-A |
| 11 | E1210RfsX8 | Stop cod1218 | c.3627insA | 3746insA | D | 1 | *BRCA1* (AB21) |  | L-A, As [52] |
| **11** | **S1253X** | **p.Ser1253Stop** | **c.3758_3759delCT** | **1372delCT** | **NR** | **1** | *BRCA1* **(AB17)** |  | **Argentina** |
| 17 | T1677IfsX2 | Stop cod 1678 | c.5030_5033delCTAA | 5149delCTAA | D | 1 | *BRCA1* (AB79) |  | E |
| 20 | S1755PfsX75 | Stop cod 1829 | c.5263insC | 5382insC | D | 2 | *BRCA1* (AB55, AB97) |  | E [53], Ashkenazi |
| ***Missense*** | | | | | | | |  |  |
| 2 | R7C | p.Arg7Cys | c.19C>T | 138C>T | CU | 2 | - | rs144792613 | NE/ME [54] |
| 5 | C61G | p.Cys61Gly | c.181T>G | 300T>C | D | 1 | *BRCA1 (*AB75) | - | E |
| 5 | R71G | p.Arg71Gly | c.211A>G | 330A>G | D | 1 | *BRCA1* (AB64) | - | E Founder Spanish [55] |
| **7** | **V122D** | **p.Val122Asp** | **c.365T>A** | **484T>A** | **NR** | **5** | *BRCA2* **(AB66)** | **-** | **Argentina** |
| **7** | **Q139K** | **p.Gln139Lys** | **c.415C>A** | **534C>A** | **NR** | **6** | **-** | **-** | **Argentina** |
| 8 | Y179C | p.Tyr179Cys | c.536A>G | 655A>G | CU | 1 | *BRCA1* (AB80) | rs56187033 | E, A-C [56] |
| 11 | Q356R | p.Gln356Arg | c.1067A>G | 1067A>G | CU | 10 | *BRCA2* (AB78); BRCA1(AB79, AB83) | rs1799950 | A-A, L-A, E, N-A |
| 11 | F486L | p.Phe486Leu | c.1456T>C | 1575T>C | CU | 1 | *BRCA1*(AB80)* | rs55906931 | E, A-C, NE/ME |
| 11 | V525I | p.Val525Ile | c.1573G>A | 1692G>A | CU | 1 | - | rs80357273 | NA |
| 11 | N550H | p.Asn550His | c.1648A>C | 1767A>C | CU | 1 | *BRCA1*(AB80)* | rs56012641 | E, A-C, NE/ME |
| 11 | D693N | p.Asp693Asn | c.2077G>A | 2196G>A | CN | 8 | *BRCA1* (AB40) | rs4986850 | Global |
| 11 | P871L | p.Pro871Leu | c.2612C>T | 2731C>T | CN | 29 | *BRCA1* (AB80, AB84, AB85); *BRCA2* (AB65) | rs799917 | Global |
| 11 | K898E | p.Lys898Glu | c.2692A>G | 2811A>G | CU | 1 | *BRCA2* (AB66) | rs80357420 | Ashkenazi |
| 11 | M1008I | p.Met1008Ile | c.3024G>A | 3143G>A | CU | 1 | - | rs1800704 | E, L-A,Ashkenazi [56] |
| 11 | E1038G | p.Glu1038Gly | c.3113G>A | 3232G>A | CN | 33 | *BRCA1* (AB40, AB80, AB84, AB85); *BRCA2* (AB59, AB65, AB98, AB99) | rs16941 | Global |
| 11 | S1040N | p.Ser1040Asn | c.3119G>A | 3238G>A | CU | 1 | *BRCA2* (AB59) [31] | rs4986852 | E [57] |
| 11 | **D1131E** | **p.Asp1131Glu** | **c.3393C>G** | **3512C>G** | **NR** | **1** | *BRCA2* **(AB65)** | **-** | **Argentina** |
| 11 | K1183R | p.Lys1183Arg | c.3548A>G | 3667A>G | CN | 34 | *BRCA1* (AB40, AB80, AB84, AB85)*BRCA2* (AB65, AB98, AB99); | rs16942 | Global |
| 11 | I1275V | p.Ile1275Val | c.3823A>G | 3942A>G | CU | 8 | - | rs80357280 | E |
| 16 | **E1586G** | **p.Glu1586Gly** | **c.4757A>G** | **4876A>G** | **NR** | **1** | **-** | **-** | **Argentina** |
| 16 | S1613G | p.Ser1613Gly | c.4837A>G | 4956A>G | CN | 33 | *BRCA1* (AB40, AB80, AB84,); *BRCA2* (AB65, AB98, AB99) | rs1799966 | Global |
| 16 | M1652T | p.Met1652Thr | c.4955T>C | 5074T>C | CU | 1 | - | rs80356968 | E |
| ***Synonymus*** | | | | |  |  |  |  |  |
| 11 | S694S | p.Ser694Ser | c.2082C>T | 2201C>T | CN | 5 | *BRCA1* (AB40, AB82, AB84) *BRCA2* (AB99) | rs1799949 | Global |
| 11 | L771L | p.Leu771Leu | c.2311T>C | 2430 T>C | CN | 30 | *BRCA1* (AB80, AB82, AB90) *BRCA2* (AB65, AB99) | rs16940 | Global |
| 13 | S1436S | p.Ser1436Ser | c.4308T>C | 4427T>C | CN | 32 | *BRCA1* (AB40, AB82, AB84) *BRCA2* (AB99) | rs1060915 | Global |
| ***Intronic*** | | | | | | | |  |  |
| I-7 | - | - | c.441+36C>T | IVS7+36C>T | CU | 19 | *BRCA1* (AB40, AB67, AB82) | rs45569832 | As [55] |
| I-7 | - | - | c.441+37del14 | IVS7+37del14 (TTTTCTTTTTTTTT) | NR | 1 | *BRCA2* (AB67) | - | L-A [38] |
| **I-7** | **-** | **-** | **c.441+38C>T** | **IVS7+38C>T** | **NR** | **19** | *BRCA1* **(AB40, AB67, AB82)** | **-** | **Argentina** |
| I-7 | - | - | c.441+41C>T | IVS7+41C>T | CU | 19 | *BRCA1* (AB40, AB67, AB82) | rs45489593 | As |
| **I-7** | **-** | **-** | **c.441+49del15** | **IVS7+49del15** | **NR** | **4** | *BRCA1* **(AB40)** | **-** | **Argentina** |
| I-7 | - | - | c.442-34C>T | IVS7-34C>T | CN | 18 | *BRCA1* (AB84, AB85) | rs799923 | E, A-C |
| I-8 | - | - | c.548-58delT | IVS8-58delT | CN | 20 | *BRCA1* (AB90)  *BRCA2* (AB65, AB99) |  | E, A-C |
| I-14 | **-** | - | c.4485-63C>G | IVS14-63C>G | CU | 15 | *BRCA1* (AB40)  *BRCA2* (AB41, AB65, AB82) | rs8176212 | Global |
| I-16 | - | - | c.4987-68A>G | IVS16-68A>G | CU | 18 | *BRCA1* (AB82)  *BRCA2* (AB65) | rs8176234 | Global |
| **I-17** | **-** | **-** | **c.5075-33C>A** | **IVS17-33C>A** | **NR** | **1** | ***BRCA2* (AB65)** | **-** | **Argentina** |
| I-18 | - | - | c.5152+66G>A | IVS18+66G>A | CU | 6 | *BRCA2* (AB65) | rs3092994 | Global |
| **I-19** | **-** | **-** | **c.5193+6T>A** | **IVS19+6T>A** | **NR** | **2** | **-** | **-** | **Argentina** |
| **I-19** | **-** | **-** | **c.5294-22T>A** | **IVS19-22T>A** | **NR** | **1** | ***BRCA2* (AB59)** | **-** | **Argentina** |
| I-23 | **-** | - | c.5468-18T>A | IVS23-18T>A | CU | 1 | - | - | E |

***BRCA1*** genomic sequence: NG_005905.1; RNA sequence: U14680. Nucleotide numbering is based on cDNA sequence and nucleotide +1 corresponds to A of the ATG translation initiation codon. **HGVS,** Human Genome Variation Society.

**Global,** as defined in BIC or when reported in at least 3 continents ethnic groups in HapMap; **E**, European; **As**, Asian; **A** Africa; **A-A** African-American; **L-A**, Latin American Caribean; **N-A**, Native-American; **A-C** America-Caucasian; **NE/ME**, Near Eastern/Middle Eastern;

**D,** Deleterious; **CU,** Clinically Unknown; **CN,** Clinically No important**;** in *Breast Information Core database* (BIC), <http://research.nhgri.nih.gov/bic/>

In bold, novel variant not previously reported.

**Table S2**: *BRCA2* sequence variants identified in Argentinean breast/ovarian cancer cases

| ***Location***  ***Exon*** | ***Codon*** | ***HGVS***  ***Protein***  ***level*** | ***HGVS***  ***DNA level*** | ***BIC***  ***DNA level*** | ***BIC***  ***Status*** | ***N° Carrier*** | ***Occurrence***  ***(Co-occurrence deleterious Mut or in ref)*** | ***SNP id*** | ***Worldwide***  ***Occurrence*** |
| --- | --- | --- | --- | --- | --- | --- | --- | --- | --- |
| *Truncating* |  |  |  |  |  |  |  |  |  |
| 11 | N955KfsX5 | Stop cod959 | c.2808_2811delACAA | 3036delACAA | D | 1 | *BRCA1 (*AB78) | - | E, L-A |
| 11 | S1982RfsX22 | Stop cod2003 | c.5946delT | 6174delT | D | 8 | *BRCA1* (AB43,AB47, AB69,AB57  AB71,AB74,AB95,B96) | - | Ashkenazi |
| 11 | K2013X | p.Lys2013Stop | c.6037A>T | 6265A>T | D | 1 | *BRC*A1 (AB34) | - | E |
| 11 | S1882X | p.Ser1882Stop | c.5644C>G | 5872C>G | D | 1 | *BRCA2* (AB117) | - | E |
| 11 | Y1894X | Stop cod1894 | c.5909insA | 6137insA | D | 1 | *BRCA2* (AB92) | - | E |
| **14** | **E2369EfsX23** | **Stop cod2391** | **c.7105insA** | **7333insA** | **NR** | **1** | *BRCA2* ***(AB98)*** | **-** | **Argentina** |
| *Missense* |  |  |  |  |  |  |  |  |  |
| 3 | Y42C | p.Tyr42Cys | c.125A>G | 353A>G | CU | 1 | - | rs4987046 | E, L-A |
| 10 | N289H | p.Asn289His | c.865A>C | 1093A>C | CN | 5 | *BRCA2* (AB31) | rs766173 | E, As, N-A |
| 10 | H372N | p.His372Asn | c.1114C>A | 1342C>A | CN | 24 | *BRCA1* (AB40, AB80 AB85)  *BRCA2* (AB59, AB65, AB12, AB78, AB91) | rs144848 | E |
| **11** | **R858I** | **p.Arg858Ile** | **c.2578G>T** | **2806G>T** | **NR** | **1** | ***BRCA2* (AB65)** | **-** | **Argentina** |
| 11 | N991D | p.Asn991Asp | c.2971A>G | 3199A>G | CN | 4 | *BRCA2* (AB31) | rs1799944 | E |
| **11** | **H1063N** | **p.His1063Asn** | **c.3187C>A** | **3415C>A** | **NR** | **1** | **-** | **-** | **Argentina** |
| 11 | D1420Y | p.Asp1420Tyr | c.4258G>A | 4486G>A | CN | 1 | - | rs28897727 | E L-A [58] |
| 11 | M1915T | p.Met1915Thr | c.5744T>C | 5972T>C | CU | 1 | - | rs4987117 | E |
| 11 | S2098F | p.Ser2098Phe | c.6749C>T | 6521C>T | CU | 1 | - | rs80358867 | E |
| 11 | R2108H | p.Arg2108His | c.6323G>A | 6551G>A | CU | 1 | - | rs35029074 | E, L-A, A-A |
| 14 | A2466V | p.Ala2466Val | c.7469C>T | 7697C>T | CU | 37 | *BRCA1* (AB40, AB82, AB80, AB88, AB98);  *BRCA2* ( AB41, AB65, AB66, AB91, AB117) | rs169547 | L-A |
| **15** | **N2486K** | **p.Asn2486Lys** | **c.7919T>G** | **7691T>G** | **NR** | **1** | **-** | **-** | **Argentina** |
| 15 | I2490T | p.Ile2490Thr | c.7469T>C | 7697T>C | CU | 6 | - | rs11571707 | L-A |
| 18 | D2723H | p.Asp2723His | c.8169G>C | 8397G>C | CU | 1 | - | rs41293511 | E |
| 27 | I3412V | p.Ile3412Val | c.10690A>G | 10462A>G | CU | 3 | - | rs1801426 | As [59] |
| *Synonymus* |  |  |  |  |  |  |  |  |  |
| 11 | S455S | p.Ser455Ser | c.1365A>G | 1593A>G | CN | 4 | - | rs1801439 | E, As |
| 11 | K1132K | p.Lys1132Lys | c.3396A>G | 3624A>G | CN | 23 | *BRCA1* (AB6, AB84) | rs1801406 | Global |
| 11 | V1269V | p.Val1269Val | c.3807T>C | 4035T>C | CN | 26 | *BRCA1* (AB78) | rs543304 | Global |
| 11 | L1521L | p.Leu1521Leu | c.4563G>A | 4791G>A | CN | 31 | *BRCA1* (AB40), *BRCA2* (AB98, AB78) | rs206075 | A |
| 11 | V2171V | p.Val2171Val | c.6513G>C | 6741G>C | CU | 17 | *BRCA1* (AB67, AB75, AB80, AB82) | rs206076 | E, A-A NE/ME |
| 14 | S2414S | p.Ser2414Ser | c.7242T>C | 7470T>C | CN | 17 | *BRCA1* (AB67,AB75, AB80, AB82) | rs1799955 | Global |
| **26** | **I3170I** | **p.Ile3170Ile** | **c.9507T>A** | **9735T>A** | **NR** | **1** | **-** | **-** | **Argentina** |
| *Intronic* |  |  |  |  |  |  |  |  |  |
| 5’UTR | - | - | c.-26G>A | 203G>A | CN | 19 | *BRCA1* (AB40, AB75, AB80, AB82)  *BRCA2* (AB99) | rs1799943 | Global |
| **I-2** | **-** | **-** | **c.68-26** | **IVS2-26G>A** | **NR** | **1** | ***BRCA1* (AB85)** | **-** | **Argentina** |
| I-4 | - | - | c.425+67A>C | IVS4+67A>C | CU | 2 | - | rs11571610 | As |
| **I-4** | **-** | **-** | **c.425+246G>C** | **IVS4+246G>C** | **NR** | **5** | **-** | **-** | **Argentina** |
| **I-4** | **-** | **-** | **c.425+364G>C** | **IVS4+364delT** | **NR** | **4** | **-** | **-** | **Argentina** |
| I-8 | - | - | c.631+56C>T | IVS8+56C>T | CU | 21 | *BRCA1* (AB67, AB80) *BRCA2* (AB65, AB78) | - | Global |
| **I-9** | **-** | **Splice defect** | **c.793+1delG** | **IVS9+1delG** | **NR** | **1** | **-** | **-** | **Argentina** |
| I-9 | - | - | c.793+65delT | IVS9+65delT | CU | 11 | *BRCA1* (AB54, AB84, AB85)  *BRCA2* (AB65, AB78) | - | E |
| I-10 | - | - | c.1909+12delT | IVS10+12delT | CU | 38 | *BRCA1* (AB75, AB80, AB82)  *BRCA2* (AB41, AB65) | - | E |
| I-11 | - | - | c.6841+80_6841+83  delTTAA | IVS11+80delTTAA | CU | 26 | *BRCA1* (AB40, AB51, AB98, AB75, AB80, AB82) | rs11571661 | Global |
| I-16 | - | - | c.7806-12T>C | IVS16-12T>C | CN | 1 | - | rs81002875 | E |
| I-16 | - | - | c.7806-14T>C | IVS16-14T>C | CU | 31 | *BRCA1* (AB75, AB80, AB82, AB98) *BRCA2* (AB65) | - | E L-A |
| I-21 | - | - | c.8755-66T>C | IVS21-66T>C | CU | 16 | *BRCA2* (AB98, AB99) | rs4942486 | Global |
| I-24 | - | - | c.9257-16T>C | IVS24-16T>C | CU | 1 | *BRCA2* (AB85) | rs11571818 | E |
| **3'UTR** | **-** | **-** | **c.*110A>C** | **nt 10595 A>C** | **NR** | **8** | **-** | **-** | **Argentina** |

***BRCA2*** genomic sequence: NW_001838072; RNA sequence: NM_001838072

Nucleotide numbering is based on cDNA sequence and nucleotide +1 corresponds to A of the ATG translation initiation codon. **HGVS,** Human Genome Variation Society.

**Global,** as defined in BIC or when reported in at least 3 continents ethnic groups in HapMap; **E**, European; **As**, Asian; **A** Africa; **A-A** African-American; **L-A**, Latin American Caribean; **N-A**, Native-American; **A-C** America-Caucasian; **NE/ME**, Near Eastern/Middle Eastern;

**D,** Deleterious; **CU,** Clinically Unknown; **CN,** Clinically No important**;** in *Breast Information Core database* (BIC), <http://research.nhgri.nih.gov/bic/>

In bold, novel variant not previously reported.
