# Supplementary material for: BRCA1 And BRCA2 analysis of Argentinean breast/ovarian cancer patients selected for age and family history highlights a role for novel mutations of putative south-American origin
Source: Springerplus. 2012 Sep 25;1:20. doi: 10.1186/2193-1801-1-20 (PMC3725882; doi:10.1186/2193-1801-1-20)
Supplement: Supplementary file 1 — Additional file 1: Table S1:BRCA1 sequence variants identified in Argentinean breast/ovarian cancer cases [38, 52–57]. (DOC 25 KB) [file 40064_2012_24_MOESM1_ESM.doc]

Date: Jul 18, 2012

To: "angela rosaria solano" [drsolanoangela@gmail.com](mailto:drsolanoangela@gmail.com)

From: "Breast Cancer Research and Treatment (BREA)" Naresh.Santhanam@springer.com Subject: BREA-D-12-08587 - Your manuscript entitled BRCA1 and BRCA2 status in Argentinean breast/ovarian cancer patients selected for age and family history

Ref.:  Ms. No. BREA-D-12-08587
BRCA1 and BRCA2 status in Argentinean breast/ovarian cancer patients selected for age and family history
Breast Cancer Research and Treatment


Dear Dr solano,

I am sorry to inform you that the Breast Cancer Research and Treatment could not accept your manuscript for publication. However, I see that your manuscript is very well suited for the journal SpringerPlus and I would like to advise you to resubmit your manuscript there.

SpringerPlus is an Open Access journal accepting manuscripts from all disciplines of Science and publishes all that are judged to be scientifically sound by its reviewers. SpringerPlus will not reject a manuscript because it is out of scope or for its perceived importance or ability to attract citations. SpringerPlus will either accept your manuscript for publication or not, you will not be asked for additional research. All articles in SpringerPlus are freely downloadable for anyone with an internet connection, no subscription is required, and copyright remains with the authors: articles can be used without any restrictions. Please note that you do not have to do reformatting of any kind. You can find more information about the journal at www.springerplus.com.  

Please note: SpringerPlus articles are free to read, an Open Access article processing fee (APC) is charged to cover all the costs associated with the publication of your article. Many institutes have funds available for publishing costs, so do ask your institute about this. If your institute is a member of SpringerOpen or BioMed Central the fee may be covered entirely or in part. For a full list of members, please see http://www.springeropen.com/inst/. Ability to pay the APC does not affect editorial decisions, waivers can be requested and we routinely waive charges for authors from low-income countries.

If you wish to transfer your manuscript to SpringerPlus, please contact editorial@springerplus.com in the next 4 weeks. Upon receipt of your approval the SpringerPlus editorial office staff will transfer your manuscript files to SpringerPlus. Please note that you will have an opportunity to update or revise the manuscript before final submission to SpringerPlus, and that we will not resubmit the manuscript without your written approval.
In your correspondence, you are encouraged to include any available decision letters or review reports for the manuscript as these may help to speed up the review process. We would be delighted to consider your article for publication in SpringerPlus; it will immediately be sent out for peer review, handled by editors and specialists in the respective field.

If you have any questions please contact Max Haring PhD, Executive editor for SpringerPlus, at max.haring@springer.com

With kind regards,

Naresh Santhanam
JEO Assistant
Breast Cancer Research and Treatment

Reviewers' comments (if any):

we receive many more mss than we can possibly submit to peer review. the information on BRCA country by country was not felt to be sufficiently novel for BCRT. we appreciate the submission.
